# Sex Differences in Myocardial Injury: Clinical Characteristics, Outcomes, and Prognostic Implications

**DOI:** 10.3390/jcm15041439

**Published:** 2026-02-12

**Authors:** Mar Rocamora-Horrach, Óscar M. Peiró, Alfredo Bardají, German Cediel, Anna Carrasquer, Isabel Fort, José Luis Ferreiro

**Affiliations:** 1 Department of Cardiology, Joan XXIII University Hospital, 43005 Tarragona, Spain; 2 Universitat Rovira i Virgili, 43007 Tarragona, Spain; 3 Health Research Institute, Universitat Rovira i Virgili, 43007 Tarragona, Spain; 4 Clinical Laboratory, Catalan Institute of Health, Camp de Tarragona-Terres de l’Ebre, 43007 Tarragona, Spain

**Keywords:** myocardial injury, sex differences, prognosis, emergency department

## Abstract

**Background**: Myocardial injury is a known predictor of adverse outcomes. However, the impact of sex on its presentation, management, and prognosis is not fully understood. The aim of this study was to examine these differences in a tertiary hospital setting. **Methods**: We conducted a retrospective observational study of consecutive emergency department patients undergoing troponin testing from January 2012 to December 2013. Myocardial injury was classified as type 1 myocardial infarction (T1MI), type 2 myocardial infarction, or non-ischemic myocardial injury (NIMI). Clinical characteristics, management, and short- and long-term outcomes were compared by sex over a median follow-up of four years. Interaction analyses were performed to assess whether the effect of myocardial injury on outcomes differed between men and women. **Results**: Among 3620 patients, myocardial injury was more prevalent in men (31.4% vs. 25.8%; *p* < 0.001), with male sex independently associated with myocardial injury (odds ratio 1.32; 95% CI 1.11–1.58; *p* = 0.002). Risk factor profiles and electrocardiographic findings differed between sexes. NIMI was more common in women, while T1MI predominated in men (*p* < 0.001). Women with myocardial injury were less frequently hospitalized than men (63.1% vs. 74.3%, *p* < 0.001). After multivariable adjustment, long-term mortality was slightly higher in men (HR 3.39; 95% CI 2.73–4.21; *p* < 0.001), whereas women had higher adjusted risks of myocardial infarction (MI) (HR 4.11; 95% CI 2.35–7.17; *p* < 0.001) and heart failure (HF) (HR 1.78; 95% CI 1.25–2.55; *p* = 0.002). No significant interaction between sex and myocardial injury was observed for any outcome. **Conclusions**: Significant sex differences exist in myocardial injury type and prognosis. Women face increased risk of future MI and HF, whereas men have higher mortality risk. The effect of myocardial injury on long-term outcomes appears similar in both sexes.

## 1. Introduction

Myocardial injury, defined by cardiac troponin (cTn) elevations above the 99th percentile, is commonly identified in the emergency department (ED) and can arise from both ischemic and non-ischemic etiologies [1,2]. Myocardial injury has been linked to unfavourable outcomes in both hospitalized patients and seemingly healthy individuals [3]. In the ED, cTn elevations frequently occur in the absence of overt myocardial ischemia. However, any elevation in troponin has been associated with increased mortality and heart failure (HF) [4,5,6], highlighting the importance of comprehensive clinical evaluation to determine the underlying cause and its prognostic implications.

Myocardial injury is frequently categorized into three main types [1]: type 1 myocardial infarction (T1MI), resulting from atherosclerotic plaque rupture and subsequent acute thrombosis; type 2 MI (T2MI), resulting from an imbalance between myocardial oxygen supply and demand without atherosclerotic plaque rupture; and non-ischemic myocardial injury (NIMI), in which troponin elevation reflects myocardial cell damage unrelated to ischemia, as commonly noted in myocarditis, acute HF and other clinical scenarios.

Sex-based differences in acute coronary syndrome are well established [5,7]. Women frequently present with atypical symptoms [8] and may face delays in both diagnosis and treatment, potentially affecting clinical outcomes [9,10,11]. However, sex-related disparities in myocardial injury are less recognized [5,7]. Differences in underlying pathophysiology may further influence therapeutic responses and prognoses [12,13].

Therefore, the aim of this study is to investigate sex-related differences in myocardial injury among consecutive ED patients by analysing its incidence, classification, associated risk factors, clinical management, and short- and long-term outcomes.

## 2. Methods

### 2.1. Study Design and Patient Selection

This retrospective observational study included all patients admitted to the ED at our tertiary hospital in Tarragona, Spain, between 1 January 2012, and 31 December 2013, who underwent cardiac troponin I (cTnI) measurements. cTnI testing was performed at the discretion of the treating physician. For patients with multiple cTnI measurements, the highest value was used. For patients with multiple ED visits, only the first episode was considered. Consecutive patient identification was achieved through laboratory records. Patients were excluded if they lacked one-year follow-up data after the index event due to foreign nationality or residence outside our reference area. Additionally, individuals under 18 years of age and those who survived a cardiac arrest were excluded.

### 2.2. Patient Involvement

Given the retrospective and observational nature of this study, patients were not involved in its design, conducting, reporting, or dissemination.

### 2.3. Cardiac Troponin I Assay

All cTnI measurements were performed in the same laboratory via a contemporary immunoassay technique (TnI-Ultra from Siemens, Advia Centaur). According to the manufacturer, the lower detection limit was 6 ng/L. The reference range for a positive cTnI test was >39 ng/L, corresponding to the 99th percentile of a reference control group, with a coefficient of variation of <10%. A single cutoff was applied for both sexes, as the assay’s analytical sensitivity does not reliably distinguish physiological differences between men and women. Evidence from prior studies indicates that a uniform threshold maintains specificity and safety, with minimal impact on diagnostic accuracy [14].

### 2.4. Myocardial Injury

Myocardial injury was defined as a cTnI concentration above the 99th percentile upper reference limit. We classified myocardial injury into T1MI, T2MI, or NIMI [15], based on the presence (T1MI, T2MI) or absence (NIMI) of myocardial ischemia. Adjudication was performed in accordance with the Universal Definition of Myocardial Infarction [1], integrating clinical context, dynamic EKG changes, troponin kinetics, and all available supporting diagnostic data; systematic coronary angiography or advanced cardiac imaging was not required for classification. T1MI was defined as myocardial injury caused by an acute atherothrombotic coronary event. T2MI represents a heterogeneous entity characterized by an imbalance between myocardial oxygen supply and demand in the absence of plaque rupture. In these cases, to specifically differentiate T2MI from NIMI, we applied the strict diagnostic criteria proposed by Saaby et al. [16].

### 2.5. Data Collection

Demographic information, key cardiovascular risk factors, and relevant histories of both cardiovascular and non-cardiovascular disease were extracted from the medical records of all included patients. Additionally, data from the initial physical examination in the ED, electrocardiographic (ECG) findings, and key laboratory parameters—such as the estimated glomerular filtration rate, calculated using the CKD-EPI formula—were collected.

### 2.6. Post-Discharge Follow-Up and Outcomes

A four-year follow-up was conducted through a review of electronic medical records, including admission and discharge notes as well as complementary test reports, to collect data on adverse events. The primary outcome was all-cause mortality, while secondary outcomes included the incidence of HF, myocardial infarction (MI), and major adverse cardiovascular events (MACE), defined as a composite of death, MI, or hospitalization for HF.

### 2.7. Statistical Analysis

Categorical variables are presented as numbers and percentages, with comparisons conducted using chi-squared tests. Continuous data are reported as medians with interquartile ranges (IQRs) and were compared using the Mann–Whitney U test. Logistic regression analyses were performed to identify independent predictors of myocardial injury for the entire cohort and for both genders. Survival probabilities were estimated using the Kaplan–Meier method and compared with the log-rank test.

To assess whether sex and myocardial injury were independent predictors of cardiovascular events, univariable and multivariable Cox regression analyses were performed using a backward stepwise procedure. In univariate Cox regression analyses, each sex-myocardial injury subgroup was compared against the remainder of the cohort rather than against a single predefined reference category. In the multivariable analysis, clinically relevant variables that were significant in the univariable analysis were included; given the close relationship between sex and myocardial injury, these variables were modelled jointly to avoid multicollinearity, and effect modification was formally assessed by including an interaction term between sex and myocardial injury. For this study, all selected variables had a *p*-value <0.05. Therefore, the multivariable Cox regression analysis was adjusted for age, hypertension, diabetes mellitus, medical history of MI, HF, peripheral artery disease, chronic kidney disease, atrial fibrillation (AF), wide QRS and ST-segment alteration.

The proportional hazards assumption was assessed using Schoenfeld residuals, while multicollinearity was evaluated by calculating the variance inflation factor. For MI and HF-related hospitalization during follow-up, all-cause death was included as a competing risk in all analyses, and the Gray method was applied. A *p*-value <0.05 was considered statistically significant. Statistical analyses were performed using STATA 14.2 (StataCorp, College Station, TX, USA).

## 3. Results

### 3.1. Baseline Characteristics

**Figure 1:**
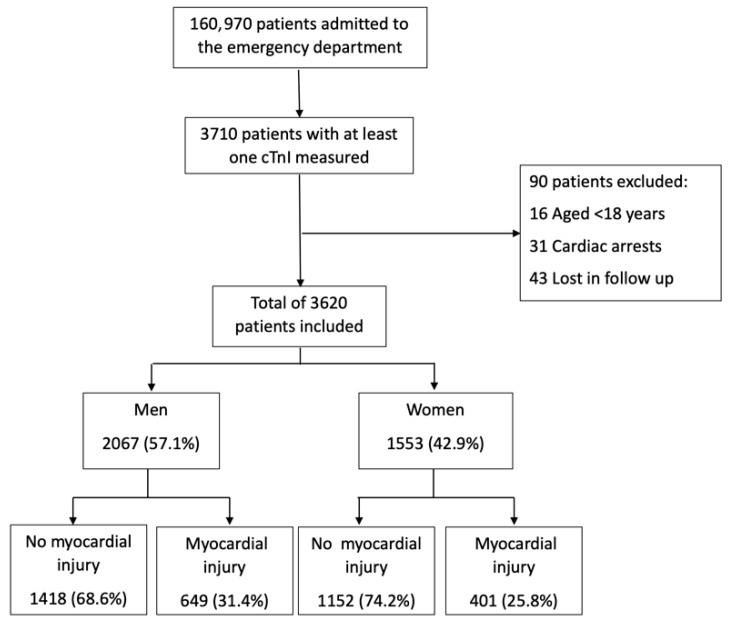
Patient flow diagram illustrating the distribution of patients by sex and presence of myocardial injury.

This study included 3620 patients, with 1553 (42.9%) being women (Figure 1). Patients with myocardial injury, in both sexes, were older, exhibited a higher prevalence of cardiovascular risk factors, and had a greater burden of prior cardiovascular disease. Furthermore, myocardial injury was more frequently observed in patients with ECG abnormalities and renal impairment. Patients with myocardial injury were more likely to require hospital admission and demonstrated a higher in-hospital mortality rate in both men and women (Table 1).

### 3.2. Sex-Based Differences in Myocardial Injury

**Figure 2:**
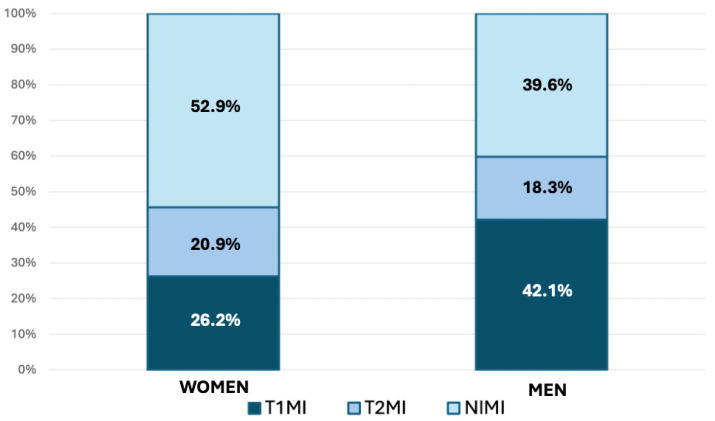
Distribution of myocardial injury types by sex.

Consistent with Figure 1, myocardial injury was significantly more prevalent in men than in women (31.4% vs. 25.8%; *p* < 0.001). Compared to men, women were older and exhibited a higher prevalence of hypertension, but were less likely to be current or former smokers. They also had a lower prevalence of prior MI and peripheral arterial disease (PAD), but a higher prevalence of HF. Chest pain was the most common presenting symptom in both sexes; however, it was less prevalent in women, who more frequently presented with dyspnea. ECG findings revealed a higher prevalence of AF in women and a higher prevalence of ST-segment alterations in men. Women were less likely to present with T1MI but more frequently presented with NIMI (Figure 2). There were no significant sex-based differences in the primary diagnoses of patients with T2MI or NIMI (Supplementary Table S1). While in-hospital mortality was similar between sexes, hospital admission was significantly lower in women.

### 3.3. Sex as a Risk Factor for Myocardial Injury

Separate analyses stratified by sex showed generally similar predictors of myocardial injury. However, hypertension and diabetes mellitus were identified as significant predictors in men, but not in women (Supplementary Table S2). In women, cerebrovascular disease, PAD, AF, and a wide QRS complex exhibited a stronger association with myocardial injury compared to men. Conversely, ST-segment abnormalities showed a stronger association with myocardial injury in men. Multivariable logistic regression analysis revealed that male sex was independently associated with myocardial injury within the entire cohort.

### 3.4. Long-Term Prognosis

**Figure 3:**
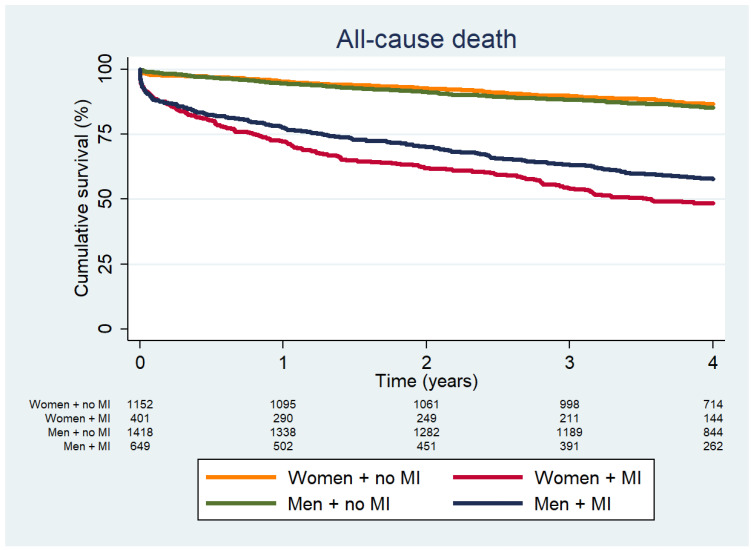
Kaplan-Meier survival curves for patients, stratified by sex and the presence or absence of myocardial injury.

During a median follow-up of 4 years (IQR 3.1–4.0), 818 patients died; 471 (44.9%) had myocardial injury, while 347 (13.5%) did not. Regardless of sex, patients with myocardial injury exhibited a significantly higher risk of long-term mortality (adjusted HR 2.77; 95% CI 2.38–3.23; *p* < 0.001; Supplementary Table S3). When stratified by sex, mortality rates were as follows: 147 (12.8%) in women without myocardial injury, 204 (50.9%) in women with myocardial injury, 200 (14.1%) in men without myocardial injury, and 267 (41.1%) in men with myocardial injury. In unadjusted analyses, women with myocardial injury had a higher risk of long-term mortality compared to men (HR 2.93; 95% CI 2.34–3.68; *p* < 0.001) (Figure 3). However, after adjusting for confounders, this difference was attenuated, with men showing a slightly higher adjusted risk (HR 3.39; 95% CI 2.73–4.21; *p* < 0.001) (Table 2). A similar trend was observed for MACE: unadjusted analyses indicated a higher risk in women with myocardial injury (HR 3.19; 95% CI 2.75–3.69; *p* < 0.001), but, after adjustment, the risk was similar in men and women (HR 2.84; 95% CI 2.35–3.44; *p* < 0.001 for men). Conversely, the adjusted hazard for MI was higher in women (HR 4.11; 95% CI 2.35–7.17; *p* < 0.001) than in men (HR 2.85; 95% CI 1.65–4.90; *p* < 0.001). Lastly, women with myocardial injury had an increased adjusted risk of long-term HF compared to men (HR 1.78; 95% CI 1.25–2.55; *p* = 0.002 vs. 1.40; 95% CI 0.98–2.01; *p* = 0.066, respectively) (Table 3).

To formally assess whether the effect of myocardial injury on outcomes differed by sex, an interaction term between sex and myocardial injury was included in the adjusted Cox models. No significant interaction was observed for all-cause mortality (*p* for interaction = 0.363). Similarly, no significant interaction between sex and myocardial injury was observed for MACE, MI, or HF (all *p* for interaction > 0.05).

## 4. Discussion

This study examined sex-related differences in myocardial injury among consecutive ED patients who underwent troponin testing and were followed for long-term outcomes. The key findings were: (1) myocardial injury was more prevalent in men, with male sex identified as an independent predictor; (2) women more frequently presented with NIMI, whereas men predominantly had T1MI; (3) despite similar hospital mortality rates within sexes, women with myocardial injury were less frequently hospitalized; (4) women with myocardial injury had a higher adjusted risk of future MI and HF than men; and (5) although unadjusted analyses suggested higher mortality and MACE risk in women with myocardial injury, these differences were attenuated after multivariable adjustment, with no evidence of a significant interaction between sex and myocardial injury on long-term outcomes.

The higher prevalence of myocardial injury in males may account for the increased utilization of troponin testing in this population [10,12]. Although prior studies have reported a greater incidence of myocardial injury in men [2,17], our finding that male sex remained independently associated with myocardial injury strengthens the existing evidence.

We observed sex-specific variations in the determinants and management of myocardial injury. Consistent with previous data [7,18], advanced age and comorbidities were strong independent predictors, and a history of cardiovascular disease was also significant in our cohort. Distinct risk factors emerged: hypertension and diabetes predicted myocardial injury in men, whereas stroke and PAD were more relevant in women. Although ECG abnormalities predict myocardial injury in both sexes [7,17], men were more likely to have ST-segment changes, while women more frequently presented with AF and a wide QRS complex. AF was treated as a potential confounder, and, after adjustment, the association between sex and myocardial injury remained significant, suggesting that AF contributes but does not fully explain the observed differences. These findings may reflect differences in the underlying pathophysiology or clinical presentation, influencing prognostic outcomes.

Sex-based disparities were also evident, with women with myocardial injury hospitalized less frequently than men, possibly reflecting differences in clinical decision-making or perceived risk [19]. In line with published data [20,21], men more often presented with T1MI, while women had higher rates of NIMI. As previously described, all types of myocardial injury were associated with worse outcomes regardless of sex [18,19], with T2MI and NIMI demonstrating poorer prognosis compared to T1MI [22].

Furthermore, unlike prior studies, our analysis included extended follow-up and evaluated a broader spectrum of outcomes beyond all-cause mortality, including MACE, MI, and HF, offering a more comprehensive assessment of the long-term consequences of myocardial injury. No cases of stress cardiomyopathy (Takotsubo) were observed, as patients with ST-segment elevation—including Takotsubo—typically bypass the ED via the STEMI pathway. Therefore, the number of T1MI patients in this study is limited to those presenting to the ED, which should be considered when interpreting the generalizability of our findings. While women with myocardial injury showed a higher adjusted risk of future MI and HF, differences in long-term mortality between sexes were attenuated after adjustment and did not reflect a significant effect modification by sex. This may partly relate to lower hospitalization rates and less intensive follow-up, potentially contributing to suboptimal risk factor control or missed opportunities for secondary prevention, and may help explain the observed differences in adjusted outcomes between men and women despite the absence of a significant interaction between sex and myocardial injury.

Overall, our study corroborates and extends prior evidence on sex differences in myocardial injury patterns and long-term outcomes, highlighting the influence of biological and clinical factors on these differences and emphasizing the need for further research to improve risk assessment and management.

### Study Limitations

This study has several limitations. While unicentric, our tertiary ED serves a population of approximately 180,000 inhabitants, providing some generalizability. Classification of MI subtypes (T1MI, T2MI, and NIMI) may be subject to misclassification; however, we applied strict criteria [16] to minimize subjectivity. Not all patients had serial troponin measurements, complicating the distinction between acute and chronic injury. Given the multiple subgroup comparisons performed in the univariate analyses, the risk of type I error is increased; accordingly, these results should be considered exploratory. The primary inferences of this study are derived from the multivariable analyses, which account for relevant confounders. The use of a single 99th percentile cTnI threshold for both sexes may have resulted in under- or over-diagnosis, potentially affecting the generalizability of our findings regarding the incidence and prognosis of myocardial injury in men and women. STEMI patients bypassed the ED and are likely underrepresented in this cohort. Culprit lesions in T1MI were identified by standard angiography, and intracoronary imaging was not systematically available. Finally, the lack of cause-specific mortality data further constrains our conclusions. These considerations underscore the need for multicenter studies with serial biomarker measurements, sex-specific thresholds, and cause-specific outcomes.

## 5. Conclusions

Significant sex differences exist in myocardial injury: women have a higher risk of future MI and HF, while men exhibit slightly higher long-term mortality. The impact of myocardial injury on outcomes is comparable across sexes, emphasizing the need for sex-informed risk assessment and management strategies in clinical practice.

## Figures and Tables

**Table 1:** Clinical characteristics in patients according to myocardial injury and sex.

	Men				Women				
Variable	Overall N = 2067	No Myocardial Injury (N = 1418)	Myocardial Injury (N = 649)	*p* Value	Overall N = 1553	No Myocardial Injury (N = 1152)	Myocardial Injury (N = 401)	*p* Value	*p* Value Myocardial Injury Men vs. Women
Demographics									
Age (years)	66.5 (52.5–78.5)	64.5 (49.5–76.5)	71.5 (58.5–81.5)	<0.001	72.5 (59.5–81.5)	68.5 (57.5–78.5)	80.5 (68.5–86.5)	<0.001	<0.001
Cardiovascular risk factors									
Current or past smoker	1003 (48.5)	623 (43.9)	380 (58.6)	<0.001	212 (13.7)	164 (14.2)	48 (12.0)	0.255	<0.001
Hypertension	1197 (57.9)	740 (52.2)	457 (70.4)	<0.001	995 (64.1)	680 (59.0)	315 (78.6)	<0.001	0.004
Diabetes mellitus	515 (24.9)	296 (20.9)	219 (33.7)	<0.001	410 (26.4)	257 (22.3)	153 (38.2)	<0.001	0.147
Medical history									
Myocardial infarction	506 (24.5)	301 (21.2)	205 (31.6)	<0.001	213 (13.7)	136 (11.8)	77 (19.2)	<0.001	<0.001
Heart failure	122 (5.9)	53 (3.7)	69 (10.6)	<0.001	135 (8.7)	70 (6.1)	65 (16.2)	<0.001	0.008
Cerebrovascular disease	174 (8.4)	94 (6.6)	80 (12.3)	<0.001	110 (7.1)	57 (5.0)	53 (13.2)	<0.001	0.673
Peripheral arterial disease	177 (8.6)	79 (5.6)	98 (15.1)	<0.001	66 (4.3)	32 (2.8)	34 (8.5)	<0.001	<0.001
Chronic kidney disease	199 (9.6)	67 (4.7)	132 (20.3)	<0.001	97 (6.3)	37 (3.2)	60 (15.0)	<0.001	0.029
Chronic pulmonary disease	411 (19.9)	261 (18.4)	150 (23.1)	0.013	240 (15.5)	157 (13.6)	83 (20.7)	<0.001	0.360
Charlson index	1 (0–3)	1 (0–2)	2 (0–4)	<0.001	1 (0–2)	1 (0–2)	2 (1–3)	<0.001	0.425
Symptoms									
Chest pain	1091 (52.8)	748 (52.8)	344 (52.9)	0.966	800 (51.5)	627 (54.4)	173 (43.1)	<0.001	0.002
Dyspnea	318 (15.4)	160 (11.3)	158 (24.4)	<0.001	288 (18.5)	165 (14.3)	123 (30.7)	<0.001	0.024
Syncope	152 (7.4)	117 (8.3)	35 (5.4)	0.021	93 (6.0)	65 (5.6)	28 (7.0)	0.330	0.292
Others	673 (32.6)	511 (36.0)	162 (25.0)	<0.001	530 (34.1)	414 (35.9)	117 (29.2)	0.015	0.133
Physical examination									
Systolic blood pressure (mmHg)	136 (120–152)	135 (120–150)	137 (119–157)	0.581	140 (124–157)	141 (125–157)	136 (120–157)	0.020	0.636
Heart rate (bpm)	78 (66–94)	76 (65–90)	83 (68–102)	<0.001	80 (67–96)	78 (67–91)	86 (69–108)	<0.001	0.066
Oxygen saturation (%)	98 (96–99)	98 (97–100)	97 (94–99)	<0.001	98 (96–100)	99 (97–100)	97 (93–99)	<0.001	0.016
ECG									
Atrial fibrillation	291 (14.9)	161 (12.1)	130 (20.9)	<0.001	281 (19.1)	159 (14.6)	122 (31.9)	<0.001	<0.001
Wide QRS	311 (15.9)	177 (13.3)	134 (21.5)	<0.001	218 (14.8)	129 (11.9)	89 (23.3)	<0.001	0.507
ST-segment alteration	329 (9.1)	85 (3.3)	244 (23.2)	<0.001	104 (6.7)	39 (3.4)	65 (16.2)	<0.001	<0.001
Negative T wave	241 (12.3)	130 (9.8)	111 (17.8)	<0.001	178 (12.1)	125 (11.5)	53 (13.9)	0.221	0.101
Laboratory tests									
Glucose (mg/dL)	112 (96–150)	107 (93–135)	131 (104–181)	<0.001	110 (94–143)	106 (93–129)	133 (103–197)	<0.001	0.535
eGFR (mL/min/1.73m2)	82 (58–97)	86 (68–99)	64 (42–88)	<0.001	76 (53–92)	80 (62–94)	55 (36–80)	<0.001	<0.001
Haemoglobin (g/dL)	14.2 (12.7–15.3)	14.4 (13.1–15.4)	13.6 (11.9–15.0)	<0.001	12.8 (11.6–13.7)	12.9 (11.9–13.8)	12.3 (11.0–13.5)	<0.001	<0.001
Maximum troponin (ng/L)	0.01 (0.01–0.08)	0.01 (0.01–0.01)	0.34 (0.09–6.19)	<0.001	0.01 (0.01–0.04)	0.01 (0.01–0.01)	0.17 (0.08–1.57)	<0.001	<0.001
Type of myocardial injury									
Type 1 AMI	273 (13.2)	-	273 (42.1)	-	105 (6.8)	-	105 (26.2)	-	<0.001
Type 2 AMI	119 (5.8)	-	119 (18.3)	-	84 (5.4)	-	84 (21.0)	-	0.298
NIMI	257 (12.4)	-	257 (39.6)	-	212 (13.7)	-	212 (52.9)	-	<0.001
Clinical evolution									
Hospital admission	746 (36.1)	264 (18.6)	482 (74.3)	<0.001	438 (28.2)	185 (16.1)	253 (63.1)	<0.001	<0.001
Hospital mortality	58 (2.8)	8 (0.6)	50 (7.7)	<0.001	45 (2.9)	15 (1.3)	30 (7.5)	<0.001	0.895

Data represent the number (percentage) or median (interquartile range). eGFR: estimated Glomerular Filtration Rate (calculated using the CKD-EPI formula). T1MI: Type 1 myocardial infarction. T2MI: Type 2 myocardial infarction. NIMI: Non-Ischemic Myocardial Injury. -, not applicable.

**Table 2:** Long-term risk of all-cause death in patients with myocardial injury.

	Univariate Cox Regression		Multivariate Cox Regression	
Variables	HR (95% CI)	*p*-Value	HR (95% CI)	*p*-Value
Age	1.08 (1.07–1.08)	<0.001	1.07 (1.06–1.07)	<0.001
Hypertension	2.58 (2.18–3.04)	<0.001	-	-
Diabetes mellitus	1.99 (1.73–2.29)	<0.001	1.27 (1.09–1.48)	0.002
Prior myocardial infarction	1.75 (1.50–2.03)	<0.001	1.15 (0.97–1.35)	0.099
Heart failure	3.35 (2.79–4.02)	<0.001	1.68 (1.38–2.05)	<0.001
Cerebrovascular disease	2.50 (2.07–3.02)	<0.001	1.37 (1.12–1.67)	0.001
Peripheral arterial disease	2.30 (1.87–2.82)	<0.001	-	-
Chronic kidney disease	3.68 (3.10–4.36)	<0.001	1.46 (1.20–1.76)	<0.001
Atrial fibrillation	2.35 (2.01–2.75)	<0.001	1.16 (0.98–1.36)	0.080
Wide QRS	1.80 (1.52–2.13)	<0.001	-	-
ST-segment alteration	1.17 (0.93–1.47)	<0.001	-	-
**Sex and myocardial injury**				
Women and no myocardial injury	0.42 (0.35–0.51)	<0.001	Reference	
Women and myocardial injury	3.37 (2.87–3.94)	<0.001	2.93 (2.34–3.68)	<0.001
Men and no myocardial injury	0.46 (0.39–0.54)	<0.001	1.33 (1.06–1.67)	0.013
Men and myocardial injury	2.64 (2.28–3.06)	<0.001	3.39 (2.73–4.21)	<0.001

Univariate and multivariate Cox regression analysis. HR: hazard ratio. CI: confidence interval. -, not applicable. Note: No significant interaction between sex and myocardial injury was observed in the adjusted Cox model (*p* for interaction = 0.363).

**Table 3:** Long-term risk of cardiovascular events by myocardial injury and sex.

	Events (%)	Unadjusted HR (95% CI); *p* Value	Adjusted HR (95% CI); *p* Value
**MACE**			
Women and no myocardial injury	204 (17.7)	0.47 (0.40–0.55); *p* < 0.001	Reference
Women and myocardial injury	234 (58.4)	3.19 (2.75–3.69); *p* < 0.001	2.66 (2.18–3.25); *p* < 0.001
Men and no myocardial injury	274 (19.3)	0.50 (0.44–0.58); *p* < 0.001	1.29 (1.07–1.56); *p* = 0.08
Men and myocardial injury	316 (48.7)	2.51 (2.20–2.86); *p* < 0.001	2.84 (2.35–3.44); *p* < 0.001
**Myocardial infarction**			
Women and no myocardial injury	21 (1.8)	0.28 (0.18–0.44); *p* < 0.001	Reference
Women and myocardial injury	38 (9.5)	2.28 (1.59–3.27); *p* < 0.001	4.11 (2.35–7.17); *p* < 0.001
Men and no myocardial injury	60 (4.2)	0.78 (0.57–1.07); *p* = 0.121	1.97 (1.20–3.23); *p* = 0.007
Men and myocardial injury	58 (8.9)	2.37 (1.73–3.24); *p* < 0.001	2.85 (1.65–4.90); *p* < 0.001
**Heart failure**			
Women and no myocardial injury	68 (5.9)	0.69 (0.53–0.91); *p* < 0.001	Reference
Women and myocardial injury	74 (18.5)	3.23 (2.47–4.23); *p* < 0.001	1.78 (1.25–2.55); *p* = 0.002
Men and no myocardial injury	54 (3.8)	0.37 (0.27–0.50); *p* < 0.001	0.77 (0.53–1.12); *p* = 0.174
Men and myocardial injury	78 (12.0)	1.87 (1.44–2.43); *p* < 0.001	1.40 (0.98–2.01); *p* = 0.066

Cardiovascular events including unadjusted and adjusted risk. HR: Hazard ratio. CI: confidence interval. MACE: Major adverse cardiovascular events (all-cause death, myocardial infarction, or hospitalization for HF). Adjusted model includes: age, hypertension, diabetes mellitus, medical history of MI, HF, peripheral artery disease, chronic kidney disease, AF, wide QRS and ST-segment alteration. Note: No significant interaction between sex and myocardial injury was observed for any of the outcomes.

## Data Availability

The datasets analyzed during the current study are available from the corresponding author upon reasonable request.

## Supplementary Materials

The following supporting information can be downloaded at https://www.mdpi.com/article/10.3390/jcm15041439/s1: Table S1: Main diagnoses of patients with type 2 myocardial infarction and non-ischaemic myocardial injury by sex; Table S2: Risk factors for myocardial injury by sex; Table S3: Long-term risk of all-cause death by myocardial injury.

## Abbreviations

The following abbreviations are used in this manuscript:

AF	atrial fibrillation
CI	confidence interval
cTn	cardiac troponin
ECG	electrocardiographic
ED	emergency department
HF	heart failure
HR	hazard ratio
IQR	interquartile range
MACE	major adverse cardiovascular events
NIMI	non-ischemic myocardial injury
PAD	peripheral arterial disease
T1MI	type 1 myocardial infarction
T2MI	type 2 myocardial infarction
