# Supplementary material for: Sex Differences in Myocardial Injury: Clinical Characteristics, Outcomes, and Prognostic Implications
Source: J Clin Med. 2026 Feb 12;15(4):1439. doi: 10.3390/jcm15041439 (PMC12942230; doi:10.3390/jcm15041439)
Supplement: Supplementary file 1 [file jcm-15-01439-s001.zip › jcm-4109816-supplementary.pdf]

Supplementary table 1: Main diagnoses of patients with type 2 myocardial infarction and non-ischaemic myocardial injury by sex.

	Overall (N=672)	Men (N=376)	Women (N=296)	P Value
Chest pain	34 (5.1)	19 (5.1)	15 (5.1)	0.993
Heart failure	138 (20.5)	69 (18.4)	69 (23.3)	0.114
Tachyarrhythmia	72 (10.7)	37 (9.8)	35 (11.8)	0.409
Bradycardia	22 (3.3)	11 (2.9)	11 (3.7)	0.567
Syncope	26 (3.9)	21 (5.6)	5 (1.7)	0.009
Anaemia	10 (1.5)	7 (1.9)	3 (1.0)	0.525
Hypertensive crisis	6 (0.9)	6 (1.6)	0 (0.0)	0.037
Pulmonary disease	109 (16.2)	63 (16.8)	46 (15.5)	0.672
Renal failure	18 (2.7)	12 (3.2)	6 (2.0)	0.353
Gastrointestinal bleeding	9 (1.3)	6 (1.6)	3 (1.0)	0.738
Other gastrointestinal pathology	31 (4.6)	13 (3.5)	18 (6.1)	0.107
Sepsis	16 (2.4)	10 (2.7)	6 (2.0)	0.593
Other infections	19 (2.8)	11 (3.7)	8 (2.1)	0.217
Neurological disease	26 (3.9)	18 (4.8)	8 (2.7)	0.164
Neoplasia	8 (1.2)	5 (1.3)	3 (1.0)	0.499
Other diagnoses	128 (19.0)	68 (18.1)	60 (20.3)	0.474

Data represent the number (percentage).

Supplementary table 2: Risk factors for myocardial injury by sex.

	**Overall** **OR (95% CI); p value**	**Men** **OR (95% CI); p value**	**Women** **OR (95% CI); p value**
Male sex	1.32 (1.11 – 1.58); 0.002	-	-
Age	1.02 (1.02 – 1.03); <0.001	1.01 (1.01 – 1.02); 0.001	1.04 (1.03 – 1.05); <0.001
Hypertension	1.36 (1.11 – 1.67); 0.003	1.41 (1.10 – 1.82); 0.008	1.20 (0.86 – 1.67); 0.292
Diabetes mellitus	1.37 (1.13 – 1.66); 0.001	1.40 (1.08 – 1.81); 0.010	1.31 (0.97 – 1.75); 0.076
Prior myocardial infarction	1.20 (0.97 – 1.47); 0.092	1.19 (0.92 – 1.54); 0.182	1.22 (0.86 – 1.74); 0.273
Heart failure	1.95 (1.44 – 2.64); <0.001	2.18 (1.41 – 3.36); <0.001	1.65 (1.08 – 2.53); 0.021
Cerebrovascular disease	1.55 (1.16 – 2.07); 0.003	1.39 (0.95 – 2.02); 0.091	1.98 (1.25 – 3.15); 0.004
Peripheral arterial disease	2.01 (1.47 – 2.76); <0.001	2.00 (1.37 – 2.94); <0.001	2.21 (1.23 – 3.95); 0.008
Chronic kidney disease	3.43 (2.55 – 4.61); <0.001	3.60 (2.50 – 5.20); <0.001	3.18 (1.91 – 5.29); <0.001
Atrial fibrillation	1.76 (1.42 – 2.19); <0.001	1.61 (1.19 – 2.18); 0.002	1.90 (1.38 – 2.62); <0.001
Wide QRS	1.74 (1.40 – 2.16); <0.001	1.56 (1.17 – 2.08); 0.002	2.01 (1.43 – 2.84); <0.001
ST segment alteration	13.67 (10.25 – 18.24); <0.001	16.92 (11.70 – 24.46); <0.001	9.44 (5.85 – 15.23); <0.001

OR indicates odds ratio; CI: confidence interval.

Supplementary table 3: Long-term risk of all-cause death by myocardial injury.

	Univariate Cox Regression		Multivariate Cox Regression	
**Variables**	**HR (95% CI)**	**P-Value**	**HR (95% CI)**	**P-Value**
Age	1.08 (1.07-1.08)	<0.001	1.07 (1.06-1.07)	<0.001
Hypertension	2.58 (2.18-3.04)	<0.001	-	-
Diabetes mellitus	1.99 (1.73-2.29)	<0.001	1.24 (1.07-1.45)	0.005
Prior myocardial infarction	1.75 (1.50-2.03)	<0.001	1.18 (1.01-1.39)	0.038
Heart failure	3.35 (2.79-4.02)	<0.001	1.70 (1.40-2.06)	<0.001
Cerebrovascular disease	2.50 (2.07-3.02)	<0.001	1.39 (1.13-1.70)	0.001
Peripheral arterial disease	2.30 (1.87-2.82)	<0.001	-	-
Chronic kidney disease	3.68 (3.10-4.36)	<0.001	1.49 (1.23-1.80)	<0.001
Atrial fibrillation	2.35 (2.01-2.75)	<0.001	-	-
Wide QRS	1.80 (1.52-2.13)	<0.001	-	-
ST segment alteration	1.17 (0.93-1.47)	<0.001	-	-
**Myocardial injury**	**4.20 (3.66-4.83)**	**<0.001**	**2.77 (2.38-3.23)**	**<0.001**

HR indicates hazard ratio; CI: confidence interval.
